# METTL10 trimethylation of eEF1A at K318 enhances protein synthesis and tumorigenesis

**DOI:** 10.1016/j.jbc.2026.113635

**Published:** 2026-10-13

**Authors:** Lauren Buie, Simone Hausmann, Juhyung Park, Sabeen Ikram, Tourkian Chasan, Xiaojie Yang, Natasha M. Flores, Pawel Mazur, Or Gozani

**Affiliations:** 1 Department of Biology, Stanford University, Stanford, California, USA; 2 Department of Experimental Radiation Oncology, The University of Texas MD Anderson Cancer Center, Houston, Texas, USA

**Keywords:** lysine methylation, protein methyltransferase, METTL10, EEF1AKMT2, eEF1A, translation elongation, KRAS, pancreatic ductal adenocarcinoma, mRNA translation

## Abstract

eEF1A is an essential non-ribosomal translation elongation factor that mediates the high-fidelity delivery of aminoacyl-tRNAs to elongating ribosomes. Like histone H3, eEF1A undergoes extensive lysine methylation, with five distinct residues targeted by dedicated lysine methyltransferases (KMTs). However, compared with histone methylation, relatively little is known about the molecular and physiological functions of eEF1A lysine methylation. Here, we focus on METTL10, the KMT that catalyzes trimethylation of eEF1A at lysine 318 (eEF1AK318me3). We show that the METTL10-eEF1AK318me3 axis modestly enhances global protein synthesis and supports pancreatic ductal adenocarcinoma (PDAC) tumorigenesis *in vivo*. We identify the 236-amino acid isoform as the catalytically active endogenous form of METTL10 and demonstrate that it is required for eEF1AK318me3 generation in multiple human cell lines. Furthermore, we provide evidence that eEF1AK318 is the principal physiologic target of METTL10. Loss of eEF1AK318me3 modestly reduces global protein synthesis across multiple cancer cell lines without substantially impacting polysome formation. METTL10 depletion attenuates proliferation of multiple cancer cell lines, an effect that depends on its catalytic activity. Moreover, elevated eEF1AK318me3 levels in PDAC tumors are associated with poor patient outcomes. Finally, METTL10 ablation attenuates tumor growth and extends survival in a KRAS^G12D^-driven PDAC mouse model. Together, our work identifies the METTL10-eEF1AK318me3 axis as a regulator of protein synthesis and implicates it in PDAC pathogenesis.

Lysine methylation is the addition of one, two, or three methyl groups to the ε-nitrogen side chain of lysine to generate mono-, di-, or trimethylated forms (1). This post-translational modification is present across human proteins and regulates diverse cellular processes ranging from chromatin biology to signal transduction 1, 2. Eukaryotic elongation factor 1A (eEF1A), an essential GTPase that delivers aminoacyl-tRNAs to the ribosome during the elongation step of mRNA translation (3), is one of the most extensively methylated protein identified in the human proteome to date 4, 5. Humans express two eEF1A paralogs: eEF1A1, which is ubiquitously expressed, and eEF1A2, whose expression is largely restricted to post-mitotic tissues (6). Notably, eEF1A1 levels are frequently elevated in tumors, whereas eEF1A2 is commonly reexpressed in cancer (6). These changes are thought to help malignant cells meet the increased demand for protein synthesis imposed by aberrant activation of growth signaling pathways, such as the RAS–MAPK and PI3K–AKT nodes 7, 8, 9. Accordingly, translation control at the level of elongation is increasingly recognized as a critical regulatory mechanism in cancer (10). Consistent with this notion, cancers with activated PI3K–AKT signaling exhibit heightened sensitivity to eEF1A inhibitors (6), highlighting the translational elongation machinery as a potential therapeutic target (10).

eEF1A is methylated at high stoichiometry at five distinct lysine residues, each generated by a dedicated seven-beta-strand (7βS) lysine methyltransferase (KMT) 4, 5. The functional consequences of eEF1A methylation remain underexplored, although some methylation events have been linked to protein synthesis regulation in physiological and/or pathological contexts. For example, di-methylation of eEF1A at lysine 55 (eEF1AK55me2) by the KMT METTL13 enhances translational output to promote tumorigenesis in KRAS-driven lung and pancreatic cancers (11). In contrast to METTL13, little is known about the biological functions of the lysine methyltransferase METTL10 (methyltransferase-like 10), which catalyzes trimethylation of eEF1A at lysine 318 (eEF1AK318me3) (12). METTL10 has been shown to be the principal KMT responsible for eEF1AK318me3 generation in two distinct human cell lines 12, 13, but whether it is broadly required for this modification across cell types and tissues or methylates additional substrates remains unclear. Further, while *METTL10* has been identified as a putative risk gene for chronic kidney disease (14), the physiological consequences of eEF1AK318me3 remain poorly understood, including whether this modification regulates protein synthesis or etiologically contributes to disease.

Here, we show that METTL10 is required for eEF1AK318me3 across multiple human cell lines and provide evidence that eEF1AK318 is its principal physiological substrate. We find that METTL10 depletion and the accompanying loss of eEF1AK318me3 reduce global protein synthesis and attenuate cancer cell proliferation, with these effects depending on METTL10 catalytic activity. Further, we find that elevated eEF1AK318me3 levels in PDAC tumor tissue samples are associated with poor patient outcomes. Finally, we find that METTL10 ablation suppresses tumor growth and extends survival in an aggressive KRAS^G12D^-driven PDAC mouse model, implicating the METTL10-eEF1AK318me3 pathway in the regulation of mRNA translation and cancer pathogenesis.

## Results

### eEF1AK318 trimethylation is the principal physiologic activity of METTL10

There are six *METTL10* transcript variants, but the physiologically relevant active isoform or isoforms have yet to be fully resolved (Figs. 1*A* and S1*A*). All six isoforms contain at least part of the predicted methyltransferase domain; however, the 158- and 213-amino acid isoforms are unlikely to be catalytically active because they lack essential structural elements of the domain, including the first β-strand, whose corresponding region is replaced by non-conserved sequence (Fig. 1*A*). In this context, it was recently demonstrated that the METTL10_236_ isoform, but not the METTL10_291_ isoform, *in vitro* methylates eEF1A at K318 (15). To investigate this further, we tested the methylation activity of the four METTL10 isoforms (METTL10_192_, METTL10_201_, METTL10_236_, and METTL10_291_) predicted to be active. To this end, we performed *in vitro* methylation assays with recombinant METTL10 isoforms, FLAG-tagged eEF1A purified from METTL10-depleted cells as a substrate lacking endogenous K318me3 (Fig. S1*B*), and tritiated *S*-adenosylmethionine (^3^H-SAM) as the methyl donor. Consistent with previous work (15), recombinant METTL10_236_ methylated eEF1A, whereas the METTL10_192_ and METTL10_201_ isoforms did not (Fig. 1*B*). While activity of the METTL10_291_ isoform was not readily detectable, prolonged exposure of reactions carried out in the presence of GTP showed a faint signal consistent with weak eEF1A methylation activity (data not shown). As previously reported (12), substitution of eEF1A K318 with arginine (eEF1A_K318R_) abolished *in vitro* methylation by METTL10_236_, consistent with K318 being the sole METTL10-dependent methylation site on eEF1A (Fig. 1*C*). We next designed an sgRNA targeting all *METTL10* isoforms (Fig. S1*C*) and analyzed control and METTL10-depleted 293T cell lysates by Western blotting with an antibody that detects all intact METTL10 isoforms (Fig. S1*D*). Depletion of METTL10 resulted in the loss of a ∼25 kDa band that co-migrated with purified recombinant METT10_236_ (Fig. 1*D*). Collectively, these data are consistent with prior work (15) and indicate that the METT10_236_ isoform is the physiologically relevant form of METTL10; hereafter, we refer to this isoform as METTL10.

**Figure 1:**
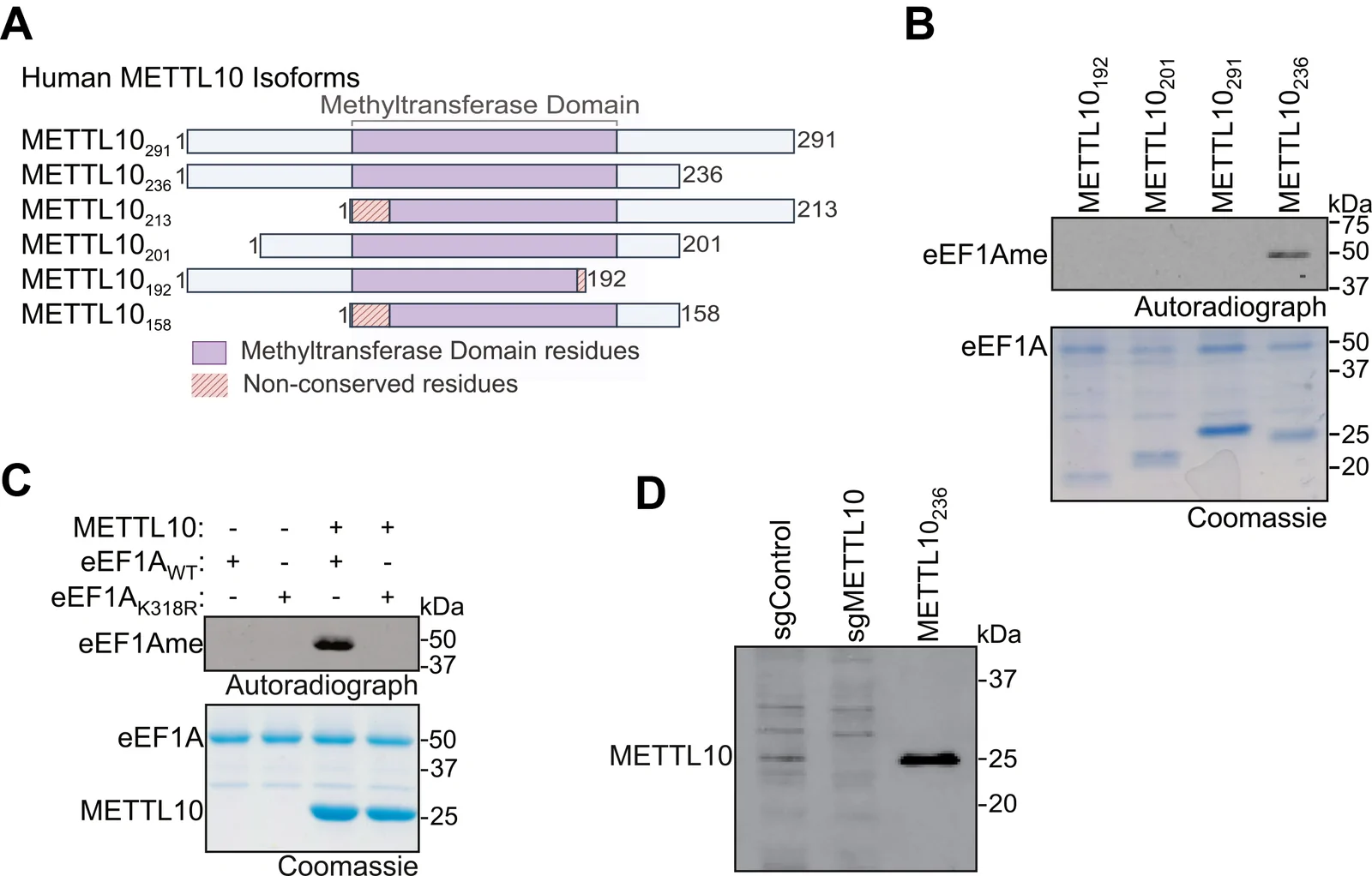
**METTL10_236_ is the catalytically active isoform in human cells.***A*, schematic representation of the protein isoforms encoded by annotated human *METTL10* transcript variants. Isoform lengths (291, 236, 213, 201, 192, and 158 amino acids [aa]) and the conserved methyltransferase (MTase) domain are indicated. *Purple shading*: residues within the MTase domain; *red hatching*: nonconserved (non-MTase) regions. *B*, *in vitro* methylation assay with the indicated recombinant METTL10 isoforms using FLAG-eEF1A lacking K318 methylation as substrate (Fig. S1*B*) and ^3^H-SAM as the methyl donor. Top panel, methylation signals detected by autoradiography. *Bottom panel*, *Coomassie stain* of proteins used in the reactions. *C*, *in vitro* methylation assay as in (*B*) using Flag-tagged wild-type or K318R eEF1A (eEF1A_WT_ and eEF1A_K318R_) as indicated. *D*, Western analysis of whole-cell extracts (WCEs) from HT1080 cells expressing sgControl or sgMETTL10, or recombinant METTL10_236_, and probed with an anti-METTL10 antibody (Fig. S1*D*) as indicated. A single band at ∼25 kDa is lost in sgMETTL10 cells and co-migrates with recombinant METTL10_236_.

Depletion of METTL10 with either of two independent sgRNAs resulted in loss of eEF1AK318me3 in the HT1080 fibrosarcoma cell line (Fig. 2*A*). This loss was also observed in the A549 lung adenocarcinoma (LUAD) and T3M4 pancreatic ductal adenocarcinoma (PDAC) cell lines (Fig. 2*B*). In all three cell lines, methylation at the other four known eEF1A lysine sites (K36, K55, K79, and K165) were intact, indicating that the direct effect of METTL10 depletion was specific to K318 methylation (Fig. 2*B*); we note that the abundance of eEF1AK79me3, a modification generated by the KMT N6AMT2 (16), may be indirectly affected by METTL10 depletion (13). Furthermore, quantitative mass spectrometry showed that eEF1AK318 is nearly fully trimethylated in wild-type cells under basal conditions and that this modification is lost upon METTL10 knockout (Figs. 2*C* and S2, *A*, *B*). Taken together, these data identify METTL10 as the enzyme responsible for eEF1AK318 trimethylation across multiple human cell lines.

**Figure 2:**
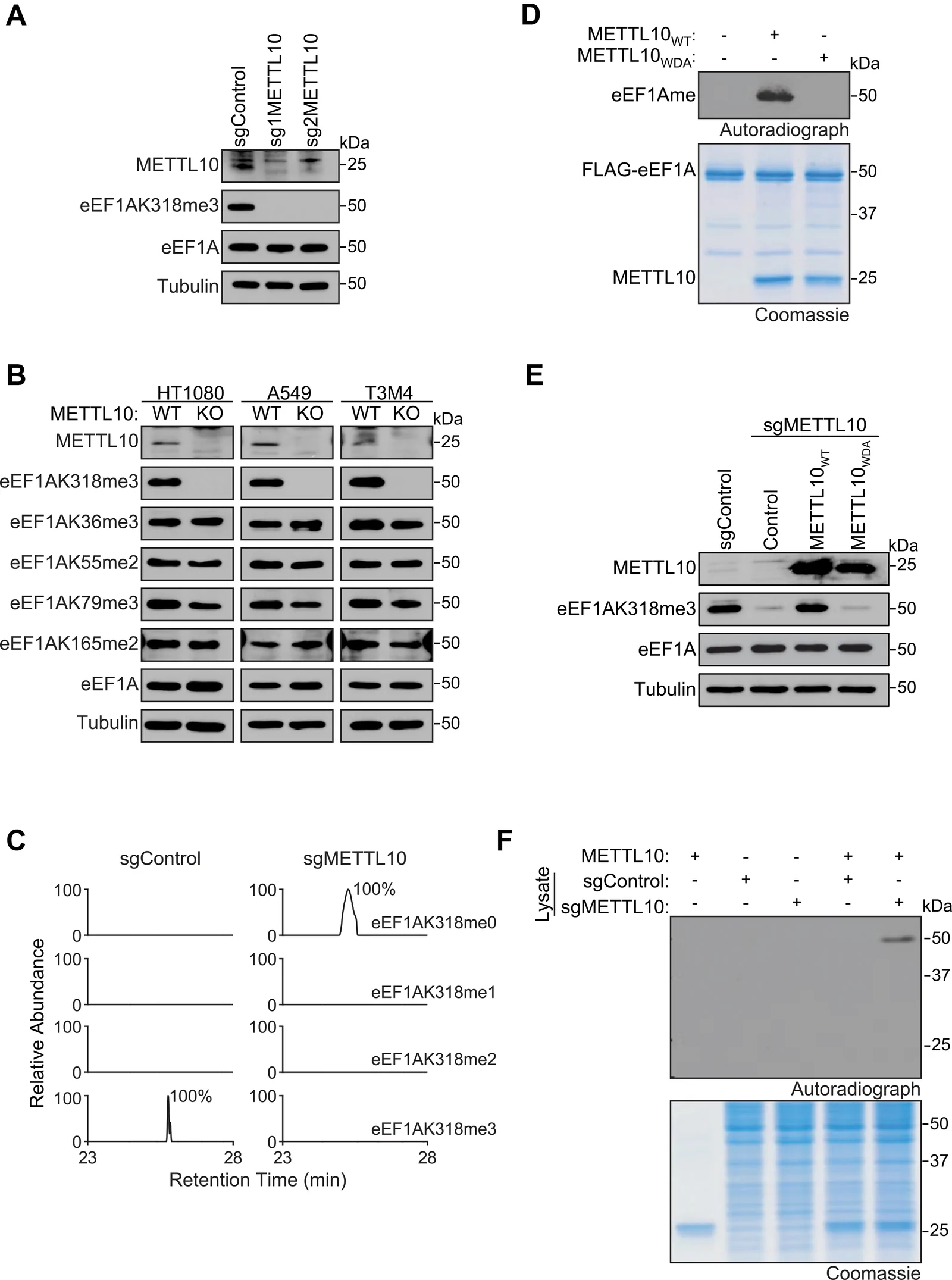
**eEF1AK318 methylation is the principal physiological activity of METTL10.***A*, Western analysis of WCEs from HT1080 cells expressing sgControl, or one of two independent sgRNAs targeting METTL10 (sg1METTL10 and sg2METTL10), probed with the indicated antibodies. Tubulin levels are shown as a loading control. *B*, METTL10 depletion selectively eliminates eEF1AK318 methylation without affecting other eEF1A lysine methylation sites. Western analysis of WCEs from the indicated cell lines expressing sgControl or sgMETTL10 using the indicated antibodies. Tubulin levels are shown as a loading control. *C*, selected ion chromatograms for unmethylated, monomethylated, dimethylated, and trimethylated eEF1AK318-containing peptides generated by GluC digestion of endogenous eEF1A immunoprecipitated from WCEs from T3M4 cells expressing sgControl or sgMETTL10, as indicated. HPLC elution profiles are shown using a 10-ppm mass window around expected m/z values for each peptide (sequence: ALPGDNVGFNVKNVSVKD, with K318 underlined; m/z = 936.994 (me0), 944.002 (me1), 951.010 (me2), and 958.018 (me3). Percentages show the relative abundance of each methylation state. *D*, *in vitro* methylation assay as in (Fig. 1*B*) using recombinant wild-type METTL10 (METTL10_WT_) or a catalytically inactive mutant bearing W59A and D109A substitutions (METTL10_WDA_), with FLAG-eEF1A as the substrate. A reaction lacking METTL10 was included as a negative control. *E*, Western analysis of WCEs from METTL10-depleted HT1080 cells complemented with CRISPR-resistant METTL10_WT_ or METTL10_WDA_ and probed with the indicated antibodies. Control: empty vector. Tubulin levels are shown as a loading control. *F*, *in vitro* methylation assay performed as in (Fig. 1*B*) using recombinant METTL10 and ^3^H-SAM as the methyl donor, with WCEs from HEK293T cells expressing sgControl or sgMETTL10 as complex substrates. Reaction products were separated by SDS-PAGE, and radiolabeled candidate substrates were detected by autoradiography. METTL10 substrates were expected to produce stronger radiolabeled bands in extracts from METTL10-depleted cells than in control extracts. A single band, migrating at the expected size of eEF1A, was detected using sgMETTL10 WCEs as the substrate; no other bands were detected.

Based on structural homology with other 7βS KMTs (17), we generated a catalytic mutant of METTL10 harboring W59A and D109A substitutions (METTL10_WDA_). Unlike wild-type METTL10, METTL10_WDA_ did not methylate eEF1A *in vitro* (Fig. 2*D*). In cells, reconstitution of METTL10-depleted A549 cells with CRISPR-resistant wild-type METTL10, but not CRISPR-resistant METTL10_WDA_, restored eEF1AK318me3 levels to control levels (Fig. 2*E*). Thus, METTL10, in a catalytic activity-dependent manner, is required for the maintenance of physiologic eEF1AK318me3 levels in cells.

Our data argue that METTL10 is the principal enzyme responsible for physiologic eEF1AK318me3. To investigate whether eEF1AK318 is likewise the principal substrate of METTL10 or one of several functionally relevant substrates, we performed *in vitro* methylation assays using recombinant METTL10, ^3^H-SAM as the methyl donor, and lysates from control or METTL10-depleted HEK293T cells as complex substrate pools (Fig. 2*F*). We used METTL10-depleted lysates to enhance substrate detection because many protein methylation sites are modified at high stoichiometry (*e.g.* 11, 13, 17, 18, 19), potentially leaving little unmethylated substrate in control lysates. We reasoned that METTL10 depletion would allow unmethylated or hypomethylated substrates to accumulate, causing candidate substrates to appear as bands unique to or more intense in the depleted lysate. A single radiolabeled band of ∼50 kDa, the molecular weight of eEF1A, was detected exclusively in reactions containing METTL10-depleted lysates; no signal was observed in control lysates. This finding is consistent with the near stoichiometric trimethylation of eEF1AK318 in wild-type cells (Fig. 2*C*; 12, 13), which leaves little unmethylated eEF1A available in control lysates for modification by recombinant METTL10. No other radiolabeled bands were detected, suggesting that METTL10 has no additional readily detectable substrates under these assay conditions. Together, these data suggest that eEF1AK318 is the principal physiologic target of METTL10, though the existence of additional substrates cannot be excluded.

### METTL10 methylation of eEF1A regulates protein synthesis

Given the fundamental role of eEF1A in translation elongation, we next tested whether METTL10-dependent methylation affects protein synthesis. METTL10 depletion modestly reduced bulk protein synthesis in A549 LUAD and PaTu8988S PDAC cells, as measured by puromycin incorporation into nascent polypeptides (Figs. 3, *A*, *B* and S2, *A*, *B*) (20). A similar reduction was observed in KP2 PDAC cells (Fig. S3*C*). This decrease was independently observed using an L-azidohomoalanine (AHA)-based assay, which monitors protein synthesis by detecting incorporation of this methionine analog into newly synthesized proteins (Figs. 3, *C*, *D* and S2, *D*, *E*) (21). Reconstitution of METTL10-depeleted A549 cells with wild-type METTL10, but not catalytically inactive METTL10_WDA_, restored protein synthesis to control levels (Fig. 3*E*). Polysome profiling of control and METTL10-depleted A549 and PaTu8988S cells showed only minor changes in ribosome distribution, with a subtle increase in the monosome fraction as the principal reproducible effect across both cell lines (Fig. 3, *F* and *G*). METTL10 depletion had similar effects on polysome profiles in MiaPaCa2 PDAC cells (Fig. S3*F*). Collectively, these data suggest that METTL10-dependent eEF1AK318me3 contributes to the regulation of global protein synthesis across multiple cell lines, although the effects are modest and appear largely uncoupled from changes in polysome formation.

**Figure 3:**
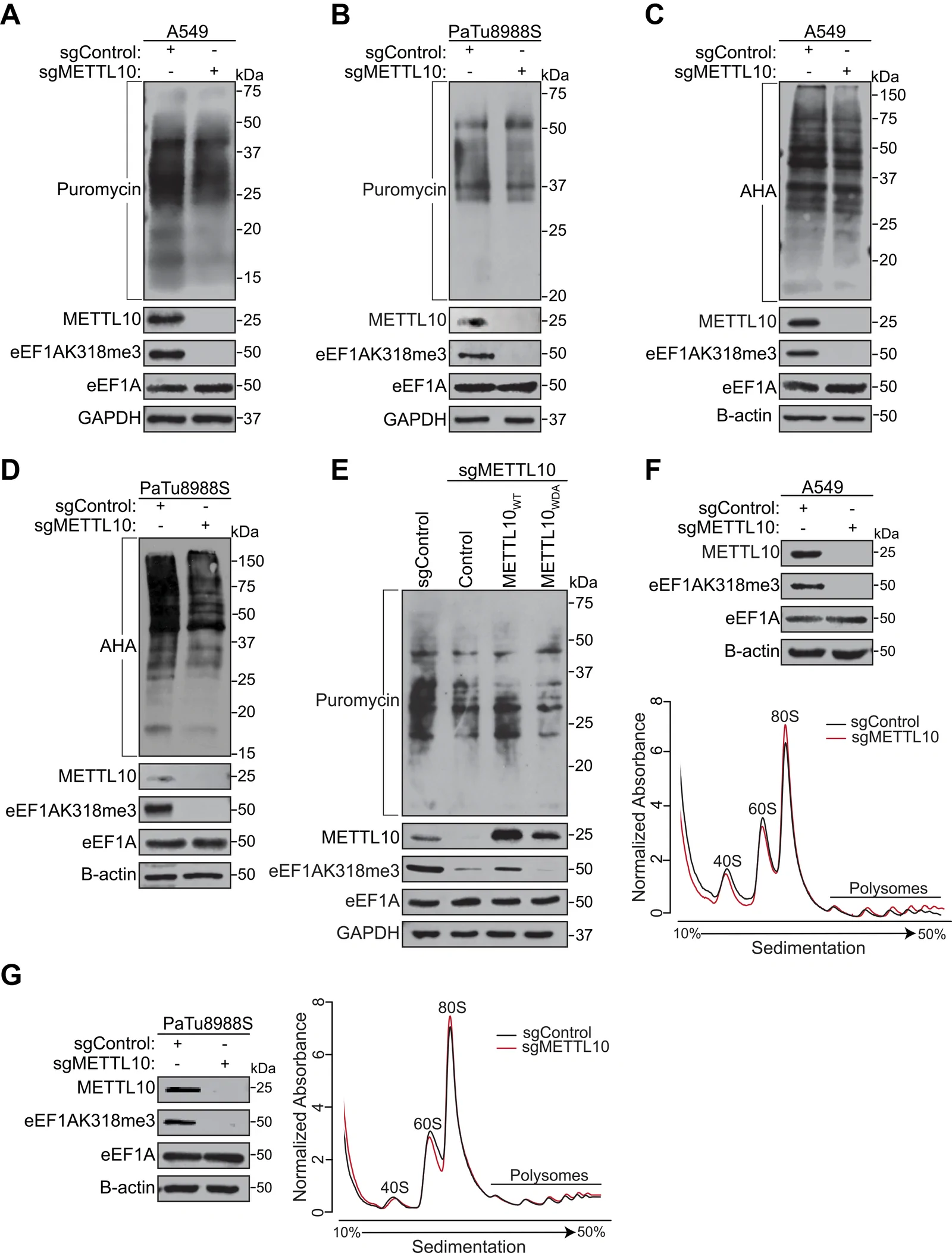
**METTL10 methylation of eEF1AK318 regulates global protein synthesis.***A-B*, Western analysis of WCEs from control or METTL10-depleted A549 (*A*) and PaTu8988S (*B*) cells probed with the indicated antibodies. Cells were pulsed for 15 min with puromycin (10 ug/ml) before harvesting. GAPDH serves as a loading control. Quantification of the puromycin signal is shown in Figure S3, *A* and *B*, respectively. *C-D*, Western analysis of WCEs from the indicated cell lines and probed with the indicated antibodies. Cells were labeled with AHA to detect newly synthesized proteins (see Methods). β-actin serves as a loading control. Quantification of the AHA signal in (*C*) and (*D*) is shown in Figure S3, *D* and *E*, respectively. *E*, METTL10-regulated protein synthesis requires its catalytic activity. Western analysis of WCEs from sgControl-treated A549 cells or METTL10-depleted A549 cells complemented with CRISPR-resistant METTL10_WT_, METTL10_WDA_, or empty vector control, and probed with the indicated antibodies. Cells were pulsed with puromycin as described in (*A*). GAPDH serves as a loading control. *F-G*, polysome profiles of control (*black*) or METTL10-depleted (*red*) A549 (*F*) and PaTu8988S (*G*) cells. Cytosolic extracts were fractionated on 10 to 50% sucrose gradients. Normalized absorbance profiles at 254 nm show the distribution of 40S and 60S ribosomal subunits, 80S monosome, and polysomes. Corresponding western analyses of WCEs from the indicated cells used for polysome profiling are shown in the top panel of (*F*) and the left panel in (*G*).

### METTL10 promotes cancer cell proliferation and tumor growth *in vivo*

Methylation of the elongation factors eEF1A and eEF2 at K55 and K525, respectively, enhances protein synthesis and promotes tumorigenesis 11, 22. We therefore tested whether METTL10 contributes to cancer cell-associated phenotypes, focusing on cellular proliferation. METTL10 depletion resulted in modest but significant reductions in the proliferation of A549 and PaTu8988S cells relative to controls (Fig. 4, *A* and *B*), with a similar effect observed in T3M4 PDAC cells (Fig. S4*A*). In METTL10-depleted A549 cells, proliferation was restored to control levels by complementation with CRISPR-resistant wild-type METTL10 but not with catalytically inactive METTL10_WDA_ (Fig. 4*C*), suggesting a role for METTL10-dependent eEF1AK318me3 in cancer cell proliferation.

**Figure 4:**
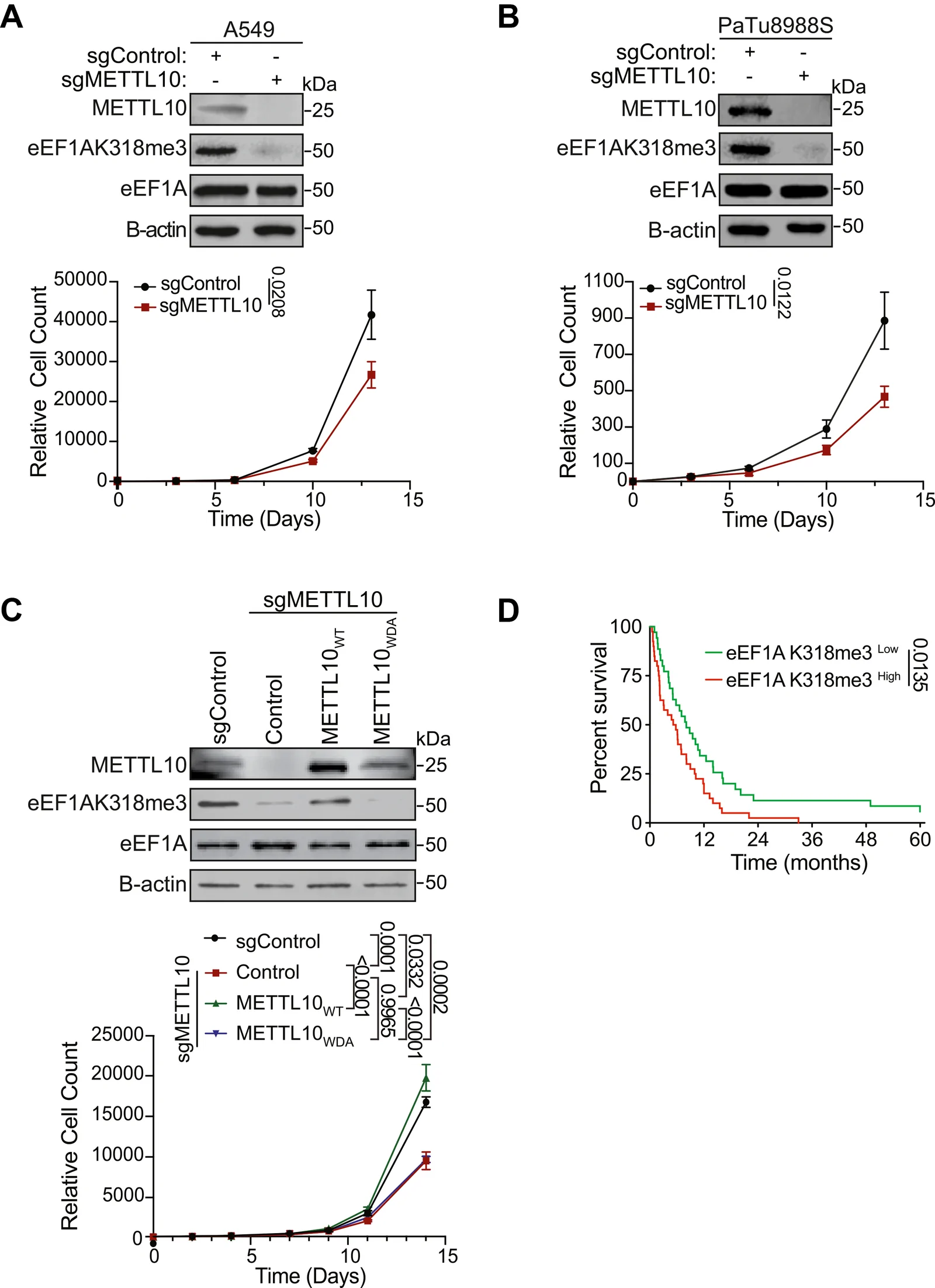
**METTL10-dependent eEF1AK318me3 contributes to cancer cell proliferation.***A-B*, METTL10 depletion attenuates the proliferation of lung and pancreatic cancer cells. Western analysis of WCEs (*top*) using the indicated antibodies and proliferation rates (*bottom*) for A549 (*A*) and PaTu8988S (*B*) cells with or without METTL10 depletion. Relative cell counts are expressed as fold change from day 0. Data represent the mean ±S.E.M. of three independent experiments. *p* values were calculated using two-tailed unpaired Student’s *t* test. *C*, METTL10-dependent proliferation of A549 cells requires its catalytic activity. Western analysis of WCEs (*top*) and proliferation rates (*bottom*) of control A549 cells or METTL10-depleted A549 cells complemented with CRISRP-resistant METTL10_WT_, METTL10_WDA_, or empty vector control, as indicated. Western blots were probed with the indicated antibodies. Relative cell counts are expressed as fold change from day 0. Data represent the mean ±S.E.M. of three independent experiments. *p* values were calculated using two-tailed unpaired Student’s *t* test. *D*, high levels of eEF1A K318me3 are associated with shorter overall survival in human PDAC. Kaplan-Meier analysis of overall survival in patients with resected PDAC (n = 74) stratified into high- and low-eEF1A K318me3 groups based on immunohistochemical chromogen intensity on a tissue microarray (see Methods). *p* value was determined by two-sided log-rank test.

Analysis of eEF1AK318me3 immunostaining in PDAC patient tissue arrays showed that elevated eEF1AK318me3 levels were associated with shorter overall survival (Fig. 4*D*). To test whether METTL10 contributes to pancreatic tumorigenesis *in vivo*, we first generated a conditional loss-of-function *Mettl10* allele (Fig. S4, *B* and *C*). We crossed *Mettl10*^LoxP^ mice with the *Kras*^LSL-G12D/+^; *Ptf1a*^Cre/+^ mice (hereafter *Kras*), a model of pancreatic neoplasia in which the *Ptf1a*^Cre^ driver directs recombination within the pancreatic epithelium 23, 24 (Fig. 5*A*). *Kras*; *Mettl10*^LoxP/LoxP^ mice (hereafter *Kras*; *Mettl10*) were born at the expected Mendelian ratios and were indistinguishable from littermate controls in body weight and general condition. These mice also showed no evidence of exocrine or endocrine insufficiency, suggesting that METTL10 is dispensable for normal pancreatic development and homeostasis (data not shown).

**Figure 5:**
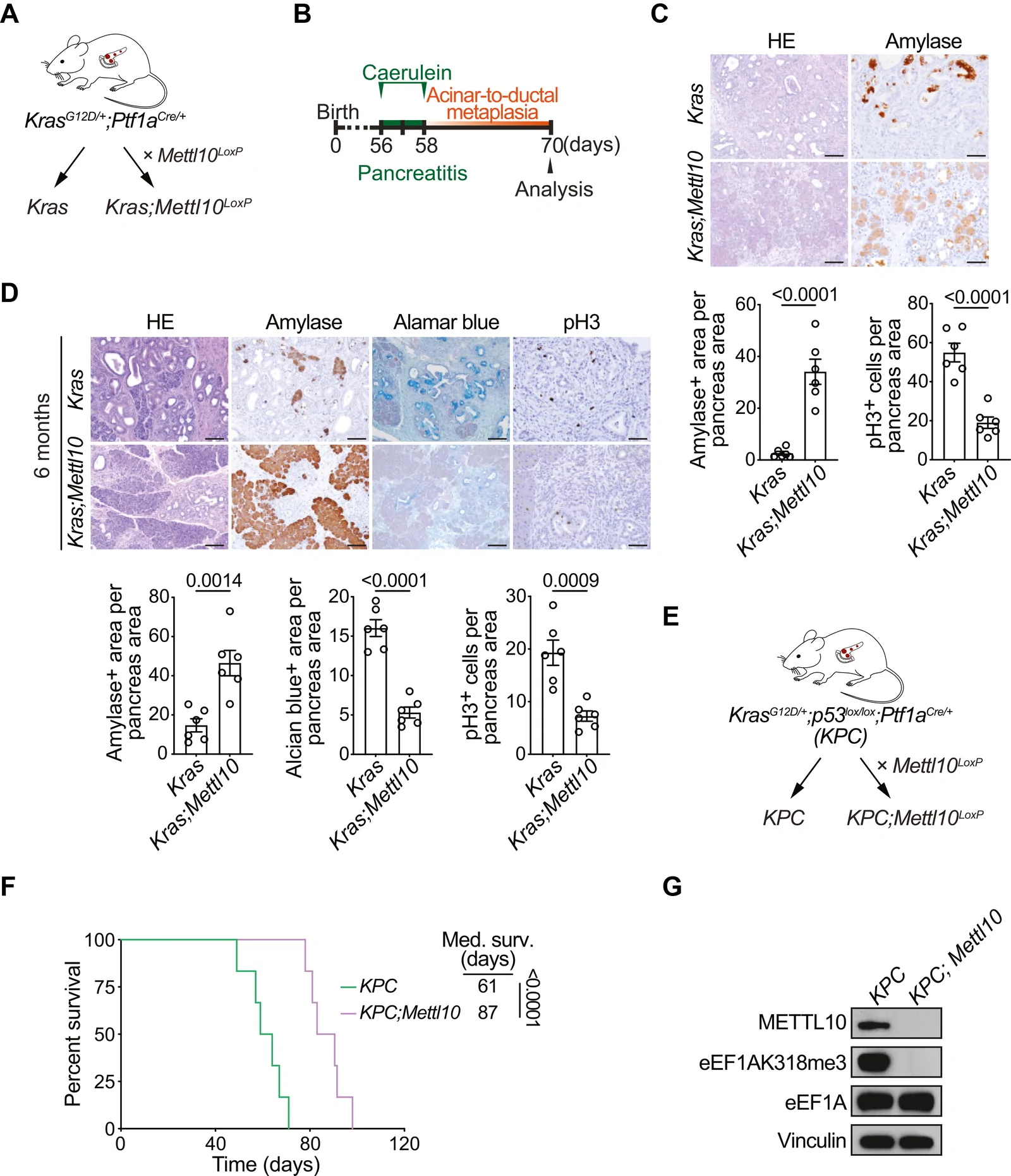
**METTL10 loss attenuates PDAC tumorigenesis *in vivo*.***A*, schematic of generation of *Kras*^*G12D/+*^*; Ptf1a*^*Cre/+*^ (*Kras*) mouse pancreatic tumorigenesis model with conditional deletion of *Mettl10* allele (*Kras; Mettl10*). Recombination of all conditional alleles is restricted to the pancreatic epithelium by the *Ptf1a*^Cre^ driver. *B*, experimental design for assessing the effect of *Mettl10* deletion on acinar-to-ductal metaplasia (ADM) as an initiating event in KRAS-driven pancreatic tumorigenesis. Pancreatitis was induced in 8-week-old mice by intraperitoneal injection of caerulein (75 μg kg^−1^) administered hourly, seven times per day, on two consecutive days. Pancreata were collected for analysis on day 70. *C*, *Mettl10* deletion preserves the acinar compartment and suppresses proliferation after caerulein-induced pancreatitis mediated pancreatic tumor initiation. *Left*: representative HE staining and IHC for amylase (marker of differentiated acinar cells) in pancreata from *Kras* and *Kras*; *Mettl10* mice harvested on day 70; scale bars, 50 μm. Right: quantification of amylase positive area (as a percentage of total pancreatic area) and of phospho-histone H3 Ser10 (pH3)^+^ cells per pancreatic area. n = 6 mice per genotype; each symbol represents one animal. *Bars* show mean ± s.e.m. *p* values determined by two-tailed unpaired Student’s *t* test. *Boxes*: 25th to 75th percentile, *whiskers*: min. to max., *center line*: median. *D*, *Mettl10* deletion suppresses spontaneous PanIN formation. *Left*: representative HE staining, Alcian blue staining (acidic mucins; PanIN marker) and IHC for amylase and pH3 in pancreata from 6-month-old *Kras* and *Kras*; *Mettl10* mice; scale bars, 50 μm. *Right*: quantification of amylase^+^ area, Alcian *blue*^+^ area (as a percentage of total pancreatic area) and pH3^+^ cells per pancreatic area. n = 6 mice per genotype; each symbol represents one animal. Bars show mean ± s.e.m. *p* values determined by two-tailed unpaired Student’s *t* test. Boxes: 25th to 75th percentile, *whiskers*: min. to max., *center line*: median. *E*, strategy for pancreas-specific deletion of *Mettl10* in the *Kras*^LSL-G12D/+^; *Trp53*^LoxP/LoxP^; *Ptf1a*^Cre/+^ (KPC) model of invasive PDAC. KPC mice were crossed to the conditional *Mettl10*^LoxP^ allele to generate KPC controls and KPC; *Mettl10*^LoxP/LoxP^ (KPC; *Mettl10*) littermates. *F*, *Mettl10* deletion extends survival in the KPC model. Kaplan-Meier survival curves of KPC (n = 6; median survival 61 days) and KPC; *Mettl10* (n = 6; median survival 87 days) mice, followed from birth to a humane endpoint defined by overall health criteria. *p* value determined by two-sided log-rank test. *G*, deletion of *Mettl10* abolishes eEF1A K318 trimethylation *in vivo*. Western analysis of representative lysates from PDAC tumors of KPC and KPC; *Mettl10* mice using the indicated antibodies. Vinculin shown as loading controls.

The development of mutant KRAS-driven PDAC is preceded by acinar-to-ductal metaplasia (ADM), a process in which acinar cells transdifferentiate into duct-like progenitors that give rise to pancreatic intraepithelial neoplasia (PanIN). ADM represents a rate-limiting step in KRAS-driven pancreatic tumor initiation 25, 26. Exocrine injury induced by acute pancreatitis accelerates ADM, providing a defined experimental window in which to test the role of METTL10 in this early step of KRAS-driven tumorigenesis. To this end, we induced pancreatitis in eight-week-old *Kras* and *Kras*; *Mettl10* mice using caerulein injections and analyzed the pancreata 12 days later (Fig. 5*B*; see Methods). At this time point, *Kras* control pancreata had undergone near-complete ADM, with the acinar parenchyma widely replaced by duct-like metaplastic structures embedded in a dense desmoplastic stroma (Fig. 5*C*). Consistent with this, amylase immunoreactivity was almost entirely lost, with amylase-positive cells occupying only ∼2% of the pancreatic area (Fig. 5*C*). In contrast, *Kras*; *Mettl10* pancreata retained large, contiguous regions of amylase^-^positive acinar tissue encompassing ∼34% of the pancreatic area, an approximately 17-fold increase relative to *Kras* controls (Fig. 5*C*). The preservation of acinar architecture was accompanied by a marked reduction in proliferation, as assessed by phospho-histone H3 staining (Fig. 5*C*). Because the proliferative burst accompanying ADM is required for the expansion of metaplastic ducts, the observed defects in ADM and proliferation are mechanistically coupled. In the absence of METTL10, KRAS^G12D^-expressing acinar cells transdifferentiate less efficiently and exhibit reduced compensatory proliferation following caerulein-induced injury. Collectively, these findings indicate that METTL10 contributes to this early step in KRAS-driven pancreatic neoplasia.

To determine whether impaired ADM translates into a reduced burden of pre-cancerous lesions, we aged *Kras* and *Kras; Mettl10* mice to 6 months, an age at which *Kras* mice reliably develop multifocal pancreatic intraepithelial neoplasia (PanIN). Consistent with the pancreatitis experiments, *Kras* pancreata showed extensively transformed regions, with numerous mucinous PanIN lesions surrounded by activated stroma. In contrast, *Kras; Mettl10* pancreata retained predominantly acinar histology and contained only scattered low-grade lesions (Fig. 5*D*). Quantification demonstrated more than a threefold increase in amylase-positive acinar area in *Kras; Mettl10* pancreata relative to *Kras* controls, indicating durable preservation of the differentiated exocrine compartment. The area positive for Alcian blue, which marks acidic mucins produced by PanIN epithelium, was reduced approximately threefold, consistent with a lower PanIN burden in pancreata lacking METTL10 (Fig. 5*D*). Proliferation in the tissue was also significantly reduced (Fig. 5*D*). These findings parallel those obtained following caerulein-induced injury and indicate that the contribution of METTL10 to KRAS-driven pancreatic transformation is not restricted to an experimentally induced inflammatory setting.

Suppression of preinvasive disease does not necessarily confer a survival benefit once tumor suppressor barriers are lost. We therefore crossed the conditional *Mettl10* allele into the *Kras*^LSL-G12D/+^; *Trp53*^LoxP/LoxP^; *Ptf1a*^Cre/+^ (KPC) model (24), in which biallelic *Trp53* loss in the context of mutant KRAS activation drives rapid progression to invasive PDAC (Fig. 5*E*). In this model, KPC control mice had a median survival of 61 days, whereas KPC; *Mettl10* mice survived for a median of 87 days, representing a ∼43% extension in median survival (Fig. 5*F*). Tumors arising in KPC; *Mettl10* mice showed complete loss of both METTL10 protein and eEF1AK318me3, indicating efficient recombination of the conditional allele (Fig. 5*G*). Together, these findings indicate that METTL10 promotes KRAS-driven PDAC tumorigenesis, while its loss impairs ADM, reduces PanIN burden, and prolongs survival in an aggressive autochthonous PDAC model without causing overt defects in normal pancreatic development.

## Discussion

Histone H3 and eEF1A are each methylated at five well-validated lysine residues, more sites than have been established for any other protein in the human proteome. On eEF1A, these modifications occur at near-saturating stoichiometry (13). Given that eEF1A is also one of the most abundant proteins in human cells, it likely constitutes one of the largest cellular reservoirs of protein lysine methylation. Despite this prevalence, and in contrast to the well-established roles of histone lysine methylation in chromatin regulation, the physiological and pathological functions of lysine methylation of the translation elongation machinery remain comparatively poorly understood. Here, we investigated the function of METTL10-catalyzed eEF1AK318me3 and found that loss of this modification modestly reduced protein synthesis and cell proliferation in lung and pancreatic cancer cell lines and impaired tumorigenesis in a KRAS-driven mouse model of LUAD. How might K318me3 mediate these effects? Structural studies place K318 on an exposed, ribosome-facing surface of eEF1A domain II, near the acceptor arm of the incoming aminoacyl-tRNA during decoding (27). This position makes a direct role in codon recognition or GTP hydrolysis unlikely. Instead, K318me3 may modulate eEF1A interactions with the ribosome or aa-tRNAs, and/or potentially influence the conformational transitions that couple codon recognition and GTPase activation to eEF1A release and tRNA accommodation. Future structural, biophysical, and functional studies will help elucidate how K318 trimethylation impacts these interactions and dynamics and, in turn, influences translation elongation and protein synthesis.

To date, there have been 16 human 7βS KMTs characterized, including METTL10 (17). DOT1L, the first member of this KMT subfamily to have its substrate activity elucidated, specifically methylates histone H3 at K79, which remains its only established target site (17). Most other characterized 7βS KMTs have likewise proven highly selective, typically modifying a single lysine residue on one protein substrate (17). For example, METTL13 follows the same selectivity principle, with K55 of eEF1A as its only established lysine target 4, 11. This one-to-one specificity of 7βS KMTs, in contrast to the broader substrate range of most enzymes that catalyze other post-translational modifications, at least in part likely arises from precise three-dimensional recognition of the substrate, and may be reinforced by the inherently slow kinetics of lysine methylation 1, 17. Although METTL10 may methylate additional physiological substrates that remain to be identified, our data indicate that K318 of eEF1A is its principal physiological target. This raises the question of why a system of five distinct KMTs evolved to modify five different lysine residues on eEF1A. We speculate that this multi-site methylation system may optimize the efficiency and adaptability of translation elongation in response to diverse cellular conditions and metabolic demands, as well as across mRNAs with different codon compositions. In future studies, it will be interesting to explore potential crosstalk among eEF1A methylation events, for example by investigating the functional and mechanistic relationship between K318me3 and K55me2. Overall, our work identifies a role for the METTL10-eEF1AK318me3 axis in regulating protein synthesis and links this pathway to cancer pathogenesis.

## Experimental procedures

### Cell lines

293T (American Type Culture Collection (ATCC), CRL-3216, female, embryonic kidney), A549 (ATCC, CCL-185, male, 58 years old, lung adenocarcinoma), HT1080 (ATCC, CCL-121, male, 35 years old, fibrosarcoma), PaTu8988S (DSMZ, ACC 204, female, 64 years old, PDAC), and MiaPaCa2 (ATCC, CRL-1420, male, 65 years old, PDAC) were cultured in Dulbecco’s modified Eagle’s medium (DMEM, Gibco) supplemented with 10% fetal bovine serum (FBS) and 1% penicillin–streptomycin. T3M4 (RIKEN, RCB1021, male, 64 years old, PDAC) and KP-2 (HSRRB, JCRB0181, female, 65 years old, PDAC) cells were cultured in RPMI 1640 medium (Gibco) supplemented with 10% FBS, 2 mM L-glutamine, and 1% penicillin–streptomycin. All cells were cultured at 37 °C in a humidified incubator with 5% CO_2_. All lines were used at low passage from authenticated repository stocks (each repository authenticates by STR profiling) and tested negative for *mycoplasma* by PCR.

### Plasmids

Protein sequences used were METTL10 (NP_001403172) and eEF1A1 (NP_001393.1); accessions for all six annotated METTL10 isoforms are listed in Figure S1*C*. Unless otherwise indicated, “METTL10” refers to the 236-amino-acid isoform (NP_001403172). For bacterial expression, coding sequences were cloned into pGEX-6P-1. For transient mammalian expression, constructs were cloned into pcDNA3.1(+) (C-terminal 1 × FLAG-tagged). For CRISPR–Cas9 knockout, the vectors lentiCRISPRv2-Hygro and lentiCRISPRv2-Blast were used. For stable reconstitution and expression in mammalian cells, the plasmid pLenti6.2 V5-DEST/blasticidin was used to express wild-type or mutant METTL10. Single-point mutations in METTL10 (*e.g.*, W59A, D109A) and eEF1A (K318R) were generated using PfuTurbo site-directed mutagenesis (Agilent) according to the manufacturer’s recommendations.

### Transfection and viral transduction

Transient expression was performed using polyethylenimine (PEI) at a ratio of 3 μg of PEI per 1 μg of plasmid DNA. For CRISPR–Cas9 knockout experiments, lentiviral particles were generated by co-transfecting 293T cells with the lentiCRISPRv2-Blast or lentiCRISPRv2-Hygro vector containing the indicated sgRNA, psPAX2, and pMD2.G plasmids at a mass ratio of 9:8:1 (sgRNA:psPAX2:pMD2.G). For complementation experiments, lentivirus was produced similarly using pLenti6.2 V5-DEST. At 12 h post-transfection, the medium was replaced with fresh medium containing 200 μM sodium butyrate. At 48 h, viral supernatants were filtered through a 0.45 μm filter, concentrated using Lenti-X Concentrator (Takara Bio), and flash-frozen in aliquots. Target cells were transduced with viral particles in the presence of 8 μg/ml polybrene, and the medium was replaced after 24 h. Selection was initiated 24 h later using 250 μg/ml hygromycin B or 8 μg/ml blasticidin. For FLAG-eEF1A reconstitution, cells were transiently transfected with pcDNA3.1 using PEI in Opti-MEM (Thermo Fisher Scientific).

The following guide RNA sequences (5′→3′) were used:

sgControl: GAAAACCATCCTTTAGGC.

sg1METTL10: GGAGGACGGTTTCGTCCCGT.

sg2METTL10: GTATGAATCGACTAATAAGG.

sg3METTL10: AAGTACCAACAAGTTCAACC.

### Expression and purification of recombinant proteins

GST fusion proteins were expressed in *Escherichia coli* BL21 by overnight culture at 20 °C in LB medium (10 g/l tryptone, 5 g/l yeast extract, and 10 g/l NaCl) supplemented with 0.1 mM IPTG (isopropyl 1-thio-β-D-galactopyranoside, Sigma), purified using Glutathione Sepharose 4B (GE Healthcare), and eluted in 10 mM reduced glutathione (Sigma). For the recombinant protein, cleavage of GST tags was performed with PreScission protease (GE Healthcare) overnight at 4 °C. Protein concentrations were measured using the Pierce Coomassie Plus Assay. DTT was added to the protein solution at a final concentration of 20 mM. Purification of recombinant METTL10 isoforms and their detection by the anti-METTL10 antibody generated in-house were confirmed by Western blot (Fig. S1*D*).

For purification of FLAG-tagged eEF1A1, eEF1A1 bearing a C-terminal FLAG tag was expressed by transient transfection in wild-type or METTL10-depleted 293T cells. After 48 h of transfection, eEF1A1 was isolated from whole-cell extracts using an anti-FLAG M2 affinity gel (Sigma) according to the manufacturer’s instructions and eluted with 200 mM 3×FLAG peptide (Sigma). The resulting purified eEF1A1 was visualized by SDS-PAGE for quality control and was immediately used for enzymatic reactions.

### METTL10 antibody

METTL10-specific antibody was raised against the non-conserved N-terminal region (residues 1–79) to maximize paralog specificity. The first 79 amino acids of METTL10 were cloned into pGEX-6P-1, expressed, and purified as a GST-fusion protein. The GST tag was cleaved as described above. The METTL10 (1–79) polypeptide was conjugated to KLH and used as an immunogen to immunize rabbits. Protein conjugation, rabbit animal protocols, immunization, and antiserum production were performed by Genemed Biotechnology, Inc. Antiserum was enriched for specific METTL10 antibodies using NHS-activated high-performance columns (Sigma) following the manufacturer’s instructions. In brief, METTL10 (1–79) was immobilized in coupling buffer containing 200 mM NaHCO_3_ (pH 8.3) and 500 mM NaCl. Antiserum was diluted 10-fold in dilution buffer containing 10 mM Tris (pH 7.5) and loaded onto the column, followed by washing with 10 mM Tris (pH 8.8). Specific antibodies were collected in elution buffer containing 100 mM glycine (pH 2.5) and immediately neutralized.

### Western blot analysis

Cells were lysed by one of two methods. For direct SDS lysis, 1 × 10^6^ cells were lysed in 200 μl of SDS lysis buffer (2% SDS in Tris-HCl, pH 6.8) and boiled at 100 °C for 20 min. Alternatively, cells were lysed in RIPA buffer (50 mM Tris-HCl, pH 7.4, 150 mM NaCl, 2 mM EDTA, 0.1% SDS, 1% (v/v) Nonidet P-40) supplemented with 1 mM phenylmethylsulfonyl fluoride (PMSF) and protease inhibitor cocktail, then sonicated and centrifuged at 14,000 rpm for 20 min at 4 °C; the supernatant was recovered and protein concentration was determined using the Bio-Rad DC Protein Assay. Protein samples (2.5–30 μg) were resolved by SDS-PAGE and transferred to a PVDF membrane (0.45 μm). The following primary antibodies were used at the indicated dilutions: METTL10 (1:500), eEF1A K318me3 (1:5000), total eEF1A (clone CBP-KK1; EMD Millipore, 05-235; RRID: AB_309663; 1:5000), eEF1A K36me3 (1:1000), eEF1A K55me2 (1:10,000), eEF1A K79me3 (1:500), eEF1A K165me2 (1:500), FLAG (clone M2; Sigma-Aldrich, F1804; RRID: AB_262044; 1:5000), puromycin (clone 12D10; EMD Millipore, MABE343; RRID: AB_2566826; 1:10,000), β-actin (clone AC-15; Sigma-Aldrich, A1978; RRID: AB_476692; 1:10,000), GAPDH (clone 14C10; Cell Signaling Technology, 2118; RRID: AB_561053; 1:5000), β-tubulin (Cell Signaling Technology, 2146; RRID: AB_2210545; 1:10,000), and vinculin (clone E1E9V; Cell Signaling Technology, 13901; RRID: AB_2728768; 1:10,000). Biotinylated proteins were detected with peroxidase-conjugated streptavidin (Jackson ImmunoResearch; 1:10,000). The anti-METTL10 and eEF1A methyl-specific antibodies were generated and validated previously (13), and specificity was further confirmed here by loss of signal upon METTL10 depletion and with recombinant METTL10 proteins. Commercial antibodies were used as validated by the manufacturer, and each detected a single band at the expected molecular weight. For chemiluminescent detection, membranes were incubated with HRP-conjugated anti-mouse and anti-rabbit secondary antibodies (Jackson ImmunoResearch; 1:10,000), developed with Amersham ECL or ECL Prime, and imaged on a LI-COR Odyssey Fc. For fluorescent detection, membranes were incubated with IRDye 680RD and 800CW anti-mouse and anti-rabbit secondary antibodies (LI-COR; 1:10,000) and imaged on the fluorescence channels of the same instrument.

### *In vitro* methylation assays

*In vitro* methylation assays were performed as previously described (28). Briefly, 3 mg of purified recombinant enzyme and 3 mg of substrate were combined with methyltransferase assay buffer (50 mM Tris, pH 8.0, 20 mM KCl, 5 mM MgCl_2_, 1 mM DTT, and 10% glycerol) and either unlabeled SAM or 2 mCi of tritiated SAM (American Radiolabeled Chemicals) to a final volume of 40 ml. 40 μM AMI-1 was added to reactions using Flag-tagged proteins purified from cells to address potential PRMT contamination. The reaction mixtures were incubated overnight at 30 °C. Reactions were resolved by SDS-PAGE, followed by autoradiography, Coomassie staining, or mass spectrometry analysis. For assays with cell extracts, extracts were prepared in cell lysis buffer (50 mM Tris, pH 8.0, 150 mM NaCl, 0.2 mM EDTA, 0.1% NP-40, and 10% glycerol), clarified by centrifugation at 14,000 rpm for 10 min, and 25 mg was loaded into the reaction mixture. Reaction mixtures were incubated overnight at 30 °C. Reactions were resolved by SDS-PAGE, followed by autoradiography, Coomassie staining, or Western blot analysis.

### Mass spectrometry of endogenous eEF1A K318

T3M4 cells were transduced with Cas9 (lentiCRISPR v2-hygro vector) and either an sgRNA targeting *METTL10* (sg3METTL10) or a non-targeting control sgRNA (GAAAACCATCCTTTAGGC). Single-cell–derived clones were isolated by limiting dilution into 96-well plates, and METTL10 knockout clones were confirmed by western blotting. For immunoprecipitation of endogenous eEF1A, equal amounts of either control or METTL10 clonal knockout T3M4 whole-cell extracts (WCEs) were incubated with anti-eEF1A (CBP-KK1) at 4 °C overnight and then with protein G magnetic beads at 4 °C for 2 h. The beads were washed with cell lysis buffer at 4 °C three times and then boiled in Laemmli buffer. The immunoprecipitated eEF1A was resolved by SDS-PAGE and stained using InstantBlue Protein Stain (Expedeon). Bands were excised and destained in 50% acetonitrile, 50 mM ammonium bicarbonate (NH_4_HCO_3_) for 10 min, twice. Gel pieces were incubated in 50 mM NH_4_HCO_3_ containing 10 mM DTT at 60 °C for 30 min, followed by treatment with 25 mM iodoacetamide in 50 mM NH_4_HCO_3_ at room temperature for 45 min. In-gel digestion was performed using 100 ng/μl Glu-C in 50 mM phosphate buffer (pH 7.8) at 37 °C overnight. Two consecutive peptide extractions were performed with 5% formic acid, 49% water, and 50% acetonitrile. The resulting peptides were dried by SpeedVac, desalted using C18 StageTips (Thermo Fisher Scientific), and analyzed on an Orbitrap Eclipse Tribrid mass spectrometer at the Stanford University MS Core as described below (MS analysis). Methylation states of eEF1A K318 were manually inspected, and the methylation site was confirmed by matching the observed MS/MS fragment ion spectra to the expected fragment ions; database searching was not performed. Extracted ion chromatograms (XICs) for the non-, mono-, di-, and trimethyl peptides spanning eEF1A K318 were analyzed using Xcalibur Qual Browser v4.1 (Thermo Fisher Scientific). The settings were as follows: peptide 302 to 319 (ALPGDNVGFNVKNVSVKD, K318 is underlined) of eEF1A1(UniProt P68104), m/z of 936.994 (me0), 944.002 (me1), 951.010 (me2) and 958.018 (me3), within a 10-ppm mass tolerance. Relative abundance of each methylation state was determined by dividing its XIC peak area by the sum of the peak areas of all methylation states (me0-me3).

### Mass spectrometry

Proteolytically digested peptides were separated using an in-house pulled and packed reversed-phase analytical column (∼25 cm in length, 100 microns of I.D.), with Dr Maisch 1.9-micron C18 beads as the stationary phase. Separation was performed with an 80-min reverse-phase gradient (2–45% B, followed by a high-B wash) on an Acquity M-Class UPLC system (Waters Corporation; Milford, MA) at a flow rate of 300 nl/min. Mobile Phase A was 0.2% formic acid in water, while Mobile Phase B was 0.2% formic acid in acetonitrile. Ions were formed by electrospray ionization and analyzed on an Orbitrap Eclipse Tribrid mass spectrometer (Thermo Scientific, San Jose, CA). The mass spectrometer was operated in data-dependent acquisition mode with a 3s cycle time. Full MS scans were acquired in the Orbitrap over an *m/z* range of 400 to 1500 at a resolution of 120,000 (at *m/z* 200). Precursors with charge states of 2 to 5 were selected for fragmentation using a 1.6 *m/z* isolation window. CID fragmentation was performed at a normalized collision energy of 35%, and MS/MS spectra were acquired in the linear ion trap using the Rapid scan rate. Dynamic exclusion was set to 30 s.

### Puromycin labeling

Control or METTL10-depleted cells were seeded at 0.5 × 10^6^ cells per 6-cm dish. Puromycin incorporation was performed by incubating cells with 10 μg/ml puromycin for 15 min at 37 °C. Cells were then harvested and lysed in RIPA buffer supplemented with 1 mM PMSF and protease inhibitor cocktail. Protein concentrations were quantified, and 5 to 10 μg of total lysate was analyzed by western blotting using an anti-puromycin antibody.

### AHA labeling

Control or METTL10-depleted cells were pre-incubated in methionine-free medium for 1 h, followed by labeling with 50 μM L-azidohomoalanine (AHA) for 2 h. Cells were harvested, lysed in RIPA buffer supplemented with 1 mM PMSF and protease inhibitor cocktail, and protein concentrations were determined. Click chemistry reactions were performed using the Click Chemistry Reaction Buffer Kit (Click Chemistry Tools) according to the manufacturer’s instructions. Briefly, 100 to 200 μg of lysate was incubated with 40 μM biotin–PEG4–alkyne for 30 min. Proteins were precipitated using methanol/chloroform extraction, and 1 to 5 μg of protein was analyzed by Western blotting using streptavidin–HRP.

### Polysome profiling

Control or METTL10-depleted cells were seeded into five 15-cm Petri dishes (1 × 10^7^ cells per dish) 24 h prior to harvesting. Cells were washed in cold PBS, flash-frozen in liquid N_2_, and scraped in 300 μl of lysis buffer (60 mM Tris, pH 7.5, 100 mM KOAc, 15 mM Mg(OAc)_2_, 0.5 mM DTT, 1% Triton X-100, 5% glycerol, 100 U/ml SUPERase·In RNase inhibitor, and protease inhibitors). Lysates were incubated on ice for 15 min, then clarified by centrifugation for 10 min at 8000×*g* (4 °C). RNA content was determined by NanoDrop, and 200 μg of RNA was loaded onto 10% to 50% sucrose gradients made in 25 mM Tris, pH 7.5, 100 mM KOAc, 5 mM Mg(OAc)_2_ and prepared using a BioComp Gradient Station. Gradients were spun for 3 h in an SW41 rotor (Beckman) at 40,000 rpm and 4 °C. Gradients were analyzed at 254 nm, and fractions were collected with a BioComp Piston Gradient Fractionator.

### Proliferation assays

Cells were seeded in triplicate at a density of 400,000 cells/well in 6-well plates on day 0. Cell counts were acquired with a Countess II FL Automated Cell Counter (Thermo Fisher Scientific) at the indicated days for 10 to 14 days and graphed as fold-change relative to day 0. After each counting, the cells were maintained at a density between 400,000 and 600,000 cells/well. Trypan blue was used to stain non-viable cells.

### Animal models

*Kras*^*G12D*^, *Trp53*^*loxP/loxP*^, and *Ptf1a*^*Cre*^ mice have been described previously 23, 24, 29. The reporter-tagged insertion with conditional potential *Mettl10* (*Eef1akmt2*)^*tm1a(EUCOMM)*^ mouse strain was obtained from the European Mouse Mutant Archive repository (30). Briefly, the *Mettl10*^*tm1a(EUCOMM)*^ targeted knock-in sequence includes the Neo-LacZ cassette flanked by *Frt* sites and exon 3 sequence flanked by *loxP* sites. Founder mice were crossed with the *Rosa26*^*FlpO*^ deleter strain (31) to generate the conditional allele *Mettl10*^*loxP/loxP*^. Mice were maintained on a mixed C57BL/6;129S1 background, and littermates were systematically used as controls in all experiments. Both male and female animals were used, and no sex differences were noted. In all experiments, animals were numbered and experiments were conducted in a blinded fashion. After data collection, genotypes were revealed and animals were assigned to groups for analysis. None of the mice with the appropriate genotype were excluded from this study or used in any other experiments. All mice were co-housed with littermates (2–5 per cage) in a pathogen-free facility at a controlled temperature of 22 °C (72 °F), a humidity of 30 to 70%, and a 12 h light/12 h dark cycle (lights on 07:00–19:00), with unrestricted access to standard food and water under the supervision of veterinarians, in an AAALAC-accredited animal facility at the University of Texas MD Anderson Cancer Center (MDACC). Mouse handling and care followed the NIH Guide for the Care and Use of Laboratory Animals. All animal procedures followed the guidelines of, and were approved by, the MDACC Institutional Animal Care and Use Committee (IACUC protocol 00001636, PI: Mazur).

### Pancreatic cancer mouse models

For pancreatic cancer development studies, *Kras*^*G12D/+*^; *Ptf1a*^*Cre/+*^ (*Kras*) and *Kras*^*G12D/+*^; *Trp53*^*loxP/loxP*^; *Ptf1a*^*Cre/+*^ (KPC) mice were interbred with mice carrying the conditional *Mettl10*^*loxP*^ allele. For pancreatitis-induced acinar-to-ductal metaplasia studies, 8-week-old *Kras* and *Kras*; *Mettl10*^*loxP*^ mice were treated with caerulein (C9026; Sigma) at 75 μg/kg in saline, administered intraperitoneally hourly, 7 times per day, for 2 consecutive days. Pancreata were harvested on day 70, 12 days after the final injection. For pancreatic intraepithelial neoplasia (PanIN) progression studies, *Kras* and *Kras*; *Mettl10*^*loxP*^ mice were aged to 6 months, the age at which control animals reliably develop multifocal PanIN lesions, and pancreata were collected for histological analysis. For pancreatic ductal adenocarcinoma (PDAC) studies, KPC and KPC; *Mettl10*^*loxP*^ mice were monitored daily for body weight changes and signs of disease progression. The survival endpoint was defined by predetermined overall health criteria scoring (>15% body-weight loss, abdominal distension, hunching, reduced mobility, or reduced grooming), applied by an observer blinded to genotype. At endpoint, tumors were processed for biochemical, histological, and immunohistochemical analysis.

### Histology and immunohistochemistry

Tissue specimens were fixed in 4% buffered formalin for 24 h and stored in 70% ethanol until paraffin embedding. Sections (3 μm) were stained with hematoxylin and eosin (H&E) or used for immunostaining studies. Tissue microarray patient specimens were collected after written patient consent and in accordance with the institutional review board-approved protocols of the University of Texas MD Anderson Cancer Center (PA19-0435, PI: Mazur). The following antibodies were used at the indicated dilutions: phospho-histone H3 S10 (ABclonal, AP0002; 1:500), amylase (Cell Signaling Technology, 3796; 1:1000), and eEF1A K318me3 (1:1000). The eEF1A K318me3 antibody was generated and validated previously (13). Immunohistochemistry (IHC) was performed on formalin-fixed, paraffin-embedded (FFPE) sections using a biotin–avidin HRP conjugate method (Vectastain ABC-HRP kit, PK4000) as described previously (19). Sections were developed with DAB and counterstained with hematoxylin. For Alcian blue staining, slides were incubated for 30 min with Alcian blue solution (pH 2.5) prepared with Alcian blue 8GX (Sigma-Aldrich, A3157) in 3% acetic acid, rinsed with water, and counterstained with Nuclear Fast Red solution for 5 min. Slides were dehydrated before mounting. Images were acquired using a PreciPoint M8 microscope equipped with PointView software and quantified using ImageJ and QuPath by an observer blinded to genotype.

### Quantification and statistical analysis

Please refer to the figure legends for sample size (n) and statistical details. All values of n refer to individual mice or individual samples. Sample sizes were chosen based on previous experience with the given experiments. Cell-culture assays were performed in triplicate in two independent experiments, unless otherwise stated. Data are expressed as mean ± SEM. Differences were analyzed by two-tailed one-sample *t* test, two-tailed unpaired Student’s *t* test, one-way ANOVA with Tukey’s multiple-comparisons test, or two-sided log-rank test, as indicated in the figure legends. Analyses were performed in Prism 11 (GraphPad) or in R using RStudio.

## Data availability

All data supporting the conclusions of this article are contained within the article and its supporting information. The mass spectrometry proteomics data have been deposited to the ProteomeXchange Consortium *via* the PRIDE partner repository with the dataset identifier PXD083722 and token XDqd0kWLm0fD. Plasmids and antibodies generated in this study are available from the corresponding author upon request.

## Supporting information

This article contains supporting information.

## Conflict of interest

The authors declare that they have no conflicts of interest with the contents of this article.
