# Supplementary material for: METTL10 trimethylation of eEF1A at K318 enhances protein synthesis and tumorigenesis
Source: J Biol Chem. 2026 Oct 13;302(12):113635. doi: 10.1016/j.jbc.2026.113635 (PMC13769582; doi:10.1016/j.jbc.2026.113635)

Supplementary Figure Legends

Figure S1:

A**,** Amino-acid sequence alignment of the indicated six annotated human *METTL10* isoforms ($METTL10_{291}$, $METTL10_{236}$, $METTL10_{213}$, $METTL10_{201}$, $METTL10_{192}$ and $METTL10_{158}$). Methyltransferase-domain residues: shaded in purple; Non-conserved residues: shaded in red.

B**,** eEF1A purified from METTL10-depleted cells lacks K318 trimethylation Western analysis of WCEs from 293T cells with our without METTL10 depletion, using with the indicated antibodies.

C**,** Summary of the METTL10 isoforms targeted by the three independent sgRNAs used in the study. RefSeq and/or UniProt accessions numbers are shown. sg3METTL10 targets all six isoforms and was used unless otherwise noted.

D**,** Western analysis with αMETTL10 antibody against the indicated purified recombinant METTL10 isoforms demonstrating that the antibody detects the isoforms that contain an intact methyltransferase domain.

Figure S1

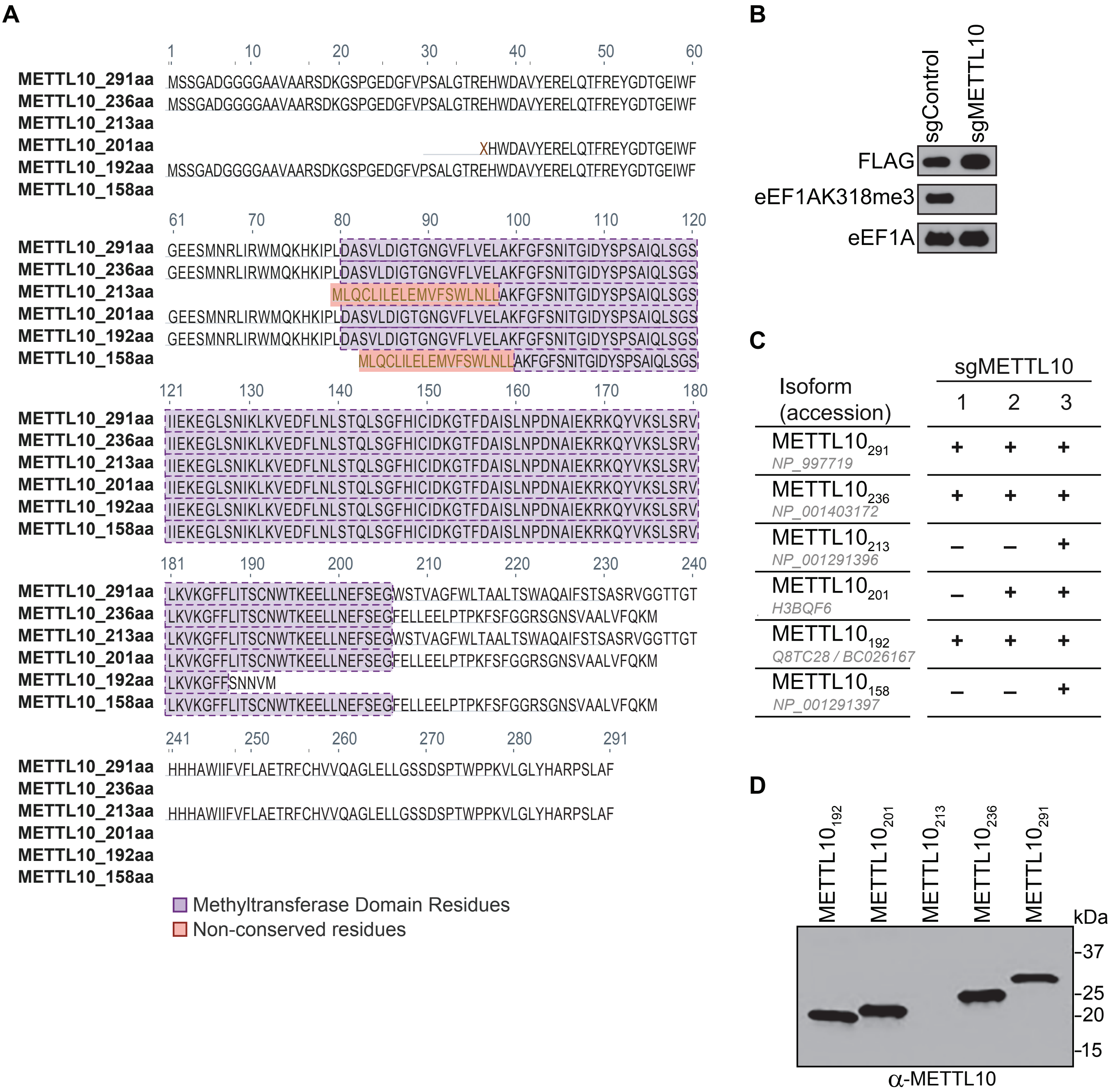
A
METTL10_291aa MSSGADGGGGAAVAARSDKGSPGEDGFVPSALGTREHWDAVYERELQTFREYGDTGEIWF
METTL10_236aa MSSGADGGGGAAVAARSDKGSPGEDGFVPSALGTREHWDAVYERELQTFREYGDTGEIWF
METTL10_213aa
METTL10_201aa XHWDAVYERELQTFREYGDTGEIWF
METTL10_192aa MSSGADGGGGAAVAARSDKGSPGEDGFVPSALGTREHWDAVYERELQTFREYGDTGEIWF
METTL10_158aa
METTL10_291aa GEESMNRLIRWMQKHKIPLDASVLDIGTGNGVFLVELAKFGFSNITGIDYSPSAIQLSGS
METTL10_236aa GEESMNRLIRWMQKHKIPLDASVLDIGTGNGVFLVELAKFGFSNITGIDYSPSAIQLSGS
METTL10_213aa MLQCLILELEMVFSWLNLLAKFGFSNITGIDYSPSAIQLSGS
METTL10_201aa GEESMNRLIRWMQKHKIPLDASVLDIGTGNGVFLVELAKFGFSNITGIDYSPSAIQLSGS
METTL10_192aa GEESMNRLIRWMQKHKIPLDASVLDIGTGNGVFLVELAKFGFSNITGIDYSPSAIQLSGS
METTL10_158aa MLQCLILELEMVFSWLNLLAKFGFSNITGIDYSPSAIQLSGS
METTL10_291aa IIEKEGLSNIKLKVEDFLNLSTQLSGFHICIDKGTFDAISLNPDNAIEKRKQYVKSLSRV
METTL10_236aa IIEKEGLSNIKLKVEDFLNLSTQLSGFHICIDKGTFDAISLNPDNAIEKRKQYVKSLSRV
METTL10_213aa IIEKEGLSNIKLKVEDFLNLSTQLSGFHICIDKGTFDAISLNPDNAIEKRKQYVKSLSRV
METTL10_201aa IIEKEGLSNIKLKVEDFLNLSTQLSGFHICIDKGTFDAISLNPDNAIEKRKQYVKSLSRV
METTL10_192aa IIEKEGLSNIKLKVEDFLNLSTQLSGFHICIDKGTFDAISLNPDNAIEKRKQYVKSLSRV
METTL10_158aa IIEKEGLSNIKLKVEDFLNLSTQLSGFHICIDKGTFDAISLNPDNAIEKRKQYVKSLSRV
METTL10_291aa LKVKGFFLITSCNWTKEELLNEFSEGWSTVAGFWLTAALTSWAQAIFSTSASRVGGTTGT
METTL10_236aa LKVKGFFLITSCNWTKEELLNEFSEGFELLEELPTPKFSFGGRSGNSVAALVFQKM
METTL10_213aa LKVKGFFLITSCNWTKEELLNEFSEGWSTVAGFWLTAALTSWAQAIFSTSASRVGGTTGT
METTL10_201aa LKVKGFFLITSCNWTKEELLNEFSEGFELLEELPTPKFSFGGRSGNSVAALVFQKM
METTL10_192aa LKVKGFFSNNVM
METTL10_158aa LKVKGFFLITSCNWTKEELLNEFSEGFELLEELPTPKFSFGGRSGNSVAALVFQKM
METTL10_291aa HHHAWIIFVFLAETRFCHVVQAGLELLGSSDSPTWPPKVLGLYHARPSLAF
METTL10_236aa
METTL10_213aa HHHAWIIFVFLAETRFCHVVQAGLELLGSSDSPTWPPKVLGLYHARPSLAF
METTL10_201aa
METTL10_192aa
METTL10_158aa
Methyltransferase Domain Residues
Non-conserved residues
B
sgControl
sgMETTL10
FLAG
eEF1AK318me3
eEF1A
C
Isoform (accession)
sgMETTL10
1 2 3
METTL10291 NP_997719 + + +
METTL10236 NP_001403172 + + +
METTL10213 NP_001291396 – – +
METTL10201 H3BQF6 – + +
METTL10192 Q8TC28 / BC026167 + + +
METTL10158 NP_001291397 – – +
D
METTL10192
METTL10201
METTL10213
METTL10236
METTL10291
kDa
37
25
20
15
α-METTL10

Figure S2:

***A*,** Representative tandem mass spectra of eEF1A K318-containing peptide shown in Fig. 2C, identifying trimethylated K318 in eEF1A isolated from control cells (top) and unmodified K318 in eEF1A isolated from METTL10-knockout cells (bottom). Assigned y and b ions are shown in red and blue, respectively. Western blot analysis of METTL10, eEF1AK318me3, eEF1A, and Tubulin levels in WCEs from control or METTL10-knockout T3M4 cells is shown in Fig. 2B.

Figure S2

A

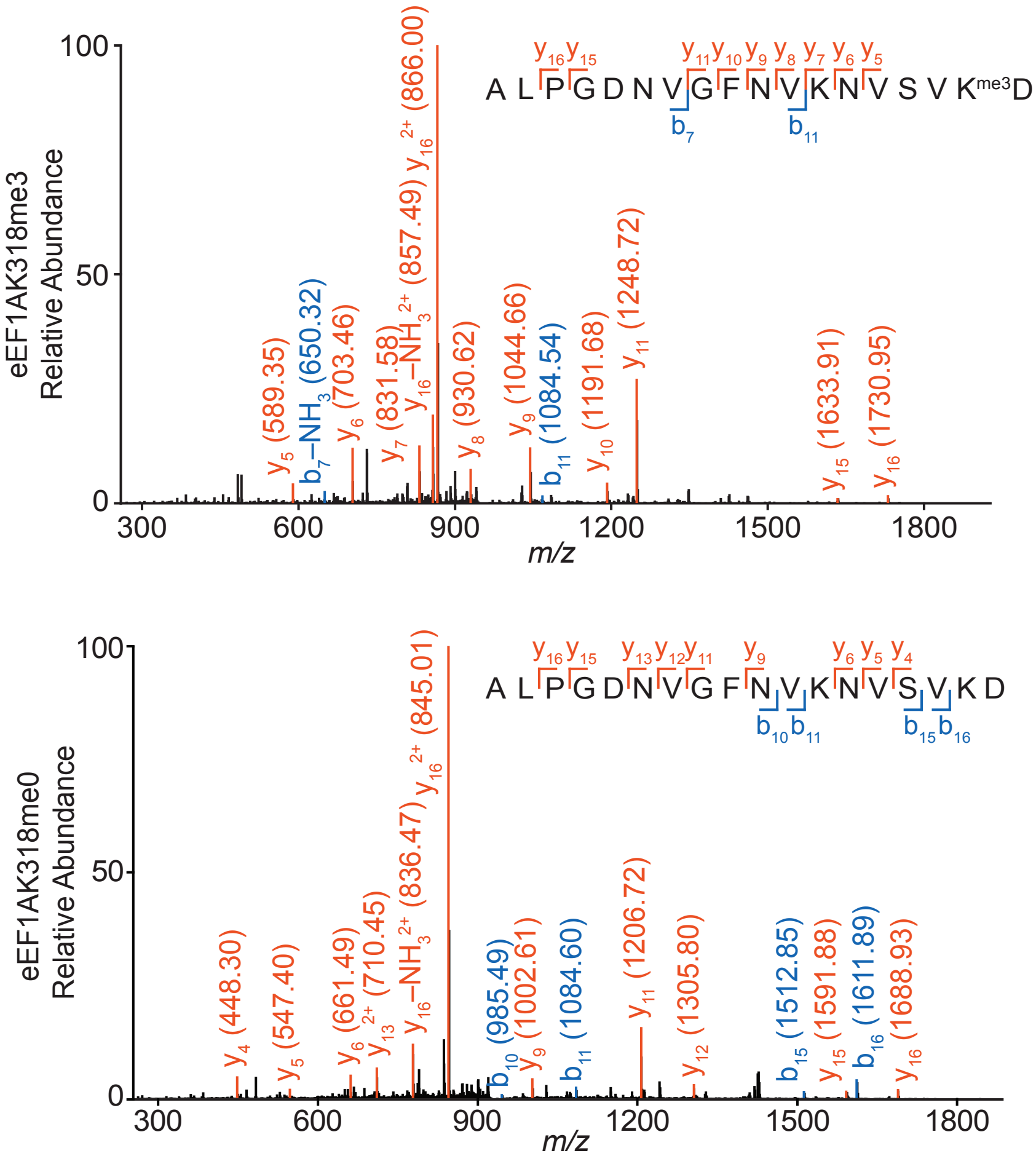

eEF1AK318me3
Relative Abundance
ALPGDNVGFNVKNVSVKme3D
y16 (866.00)
y11 (1248.72)
m/z
eEF1AK318me0
Relative Abundance
ALPGDNVGFNVKNVSVKD
y16 (845.01)
y11 (1206.72)
m/z

Figure S3:

A-B, Quantification of puromycin incorporation shown in Fig. 3A (A) and Fig. 3B (B). Data are presented as mean ± SEM from three independent replicates. *P* values determined by one-way ANOVA with Tukey's post hoc test.

C, Western analysis of WCEs from control or METTL10-depleted KP2 cells probed with the indicated antibodies. Cells were pulsed for 15 min with puromycin (10 ug/ml) before harvesting. Tubulin serves as a loading control.

D-E, Quantification of AHA labeling shown in Fig. 3C (D) and Fig. 3D (E). Data are presented as mean ± SEM from three independent replicates. *P* values determined by one-way ANOVA with Tukey's post hoc test.

F, Polysome profiles of control (black) or METTL10-depleted (red) MiaPaCa2 cells. Cytosolic extracts were fractionated on 10-50% sucrose gradients. Normalized absorbance profiles at 254 nm show the distribution of 40S and 60S ribosomal subunits, 80S monosome, and polysomes. Corresponding western analyses of WCEs from the indicated cells used for polysome profiling are shown in the left panel.

Figure S3

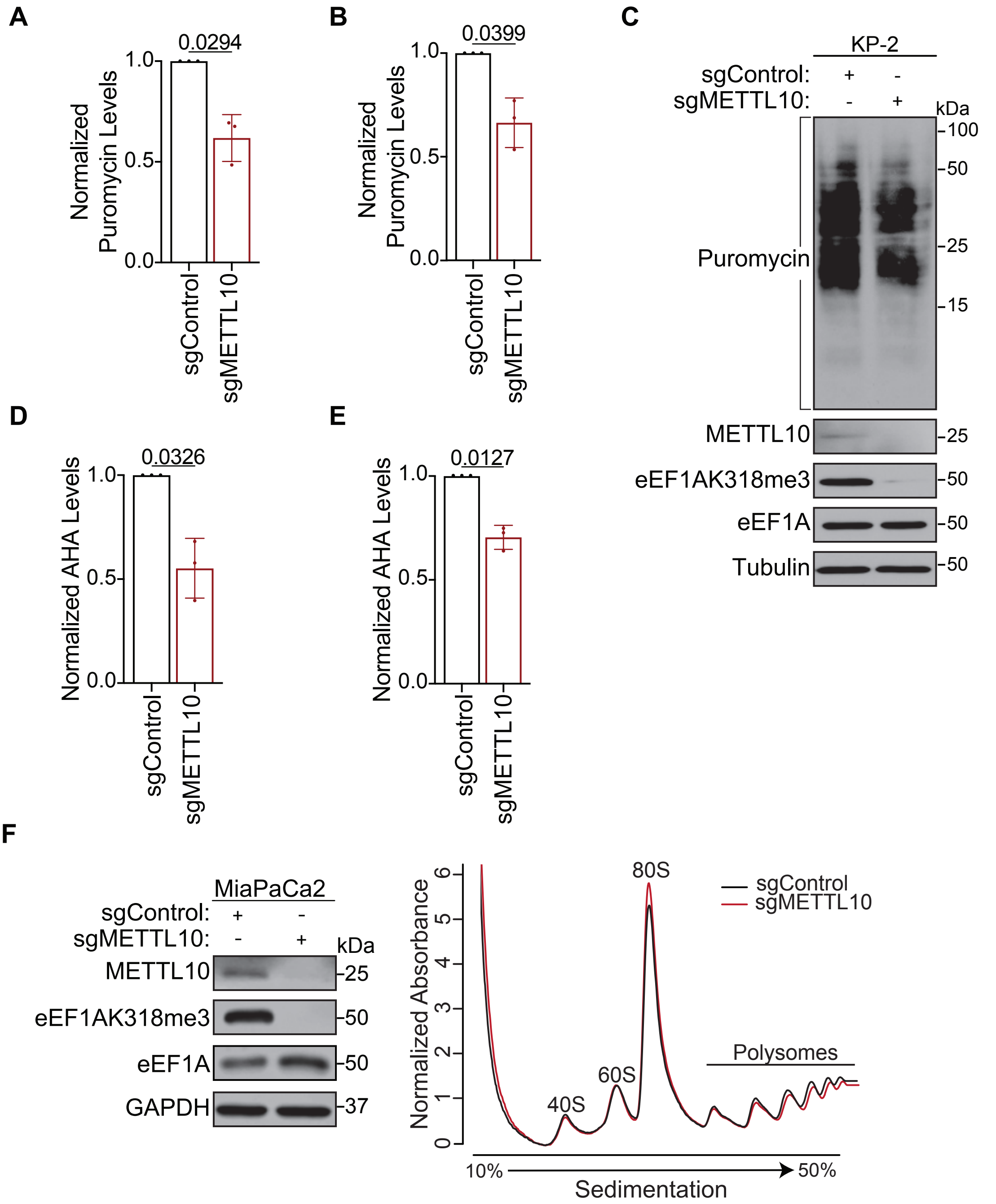

A
0.0294
Normalized Puromycin Levels
1.0
0.5
0.0
sgControl
sgMETTL10
B
0.0399
Normalized Puromycin Levels
1.0
0.5
0.0
sgControl
sgMETTL10
C
KP-2
sgControl: + -
sgMETTL10: - +
kDa
Puromycin
100
50
25
15
METTL10
25
eEF1AK318me3
50
eEF1A
50
Tubulin
50
D
0.0326
Normalized AHA Levels
1.0
0.5
0.0
sgControl
sgMETTL10
E
0.0127
Normalized AHA Levels
1.0
0.5
0.0
sgControl
sgMETTL10
F
MiaPaCa2
sgControl: + -
sgMETTL10: - +
kDa
METTL10
25
eEF1AK318me3
50
eEF1A
50
GAPDH
37
Normalized Absorbance
0
1
2
3
4
5
6
40S
60S
80S
Polysomes
sgControl
sgMETTL10
10%
50%
Sedimentation

Figure S4:

***A*,** METTL10 depletion attenuates the proliferation of T3M4 PDAC cells. Western analysis of WCEs (left) using the indicated antibodies and proliferation rates (right) for T3M4 cells expressing control sgRNA or two independent METTL10-targeting sgRNAs as indicated. Relative cell counts are expressed as fold change from day 0. Data represent the mean ±S.E.M. of three independent experiments. P values were calculated using two-tailed unpaired Student's t test.

***B*,** Schematic of the conditional *Mettl10*^{LoxP} allele. The *Mettl10*$^{\text{tm1a(EUCOMM)}}$ knockout-first allele was converted to a conditional allele by FlpO-mediated excision of the FRT-flanked Neo–LacZ cassette, leaving exon 3 flanked by LoxP sites (green triangles). In the presence of *Ptf1a*^{Cre}, exon 3 is deleted, generating a frameshift that abolishes METTL10 expression.

***C*,** Confirmation of the conditional allele by PCR genotyping of genomic DNA isolated from tail biopsies of wildtype, *Mettl10*$^{\text{LoxP/+}}$ and *Mettl10*$^{\text{LoxP/LoxP}}$ mice. Arrowheads indicate the wild-type and LoxP-containing products of the expected sizes.

Figure S4

A

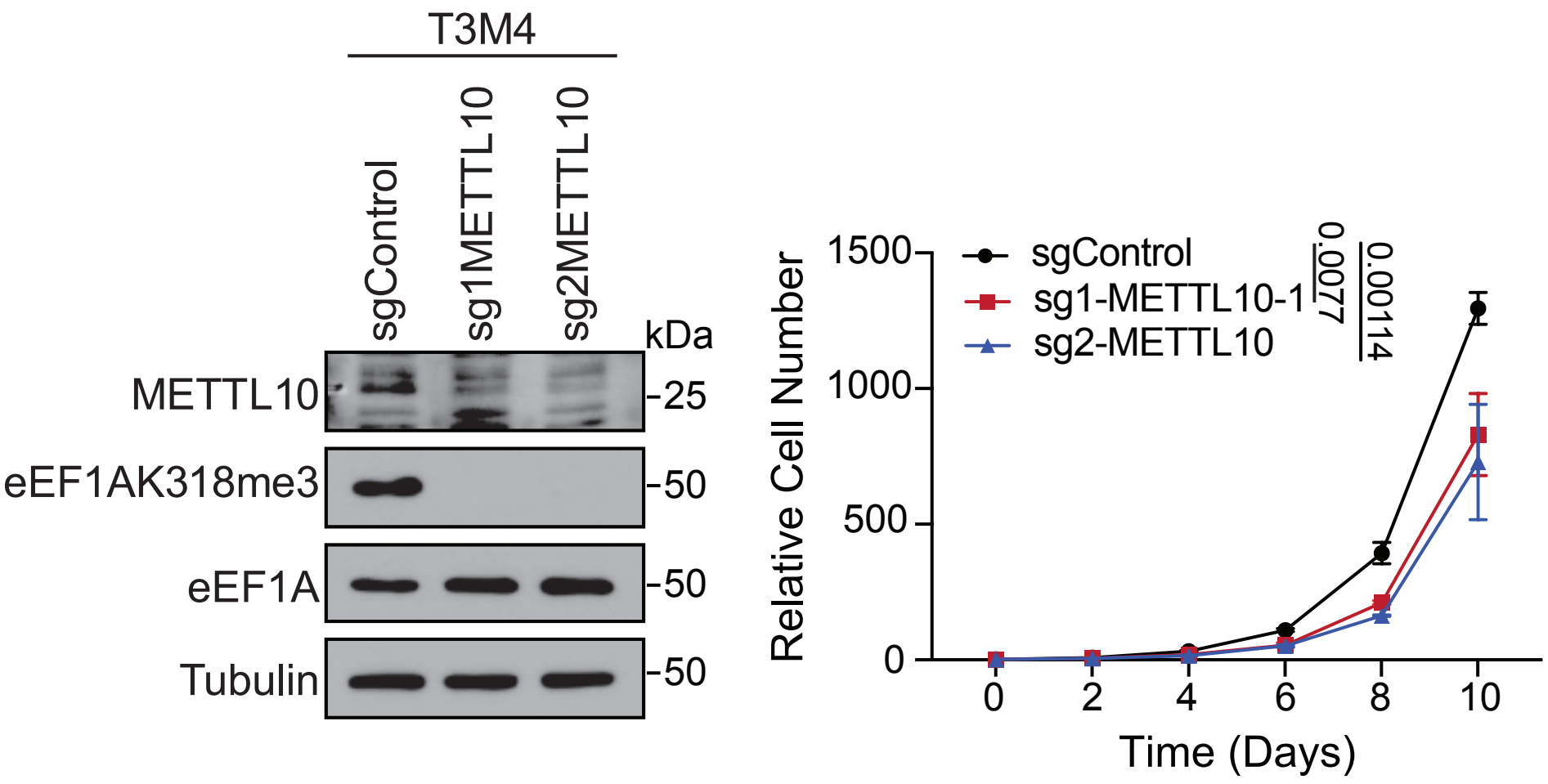

B

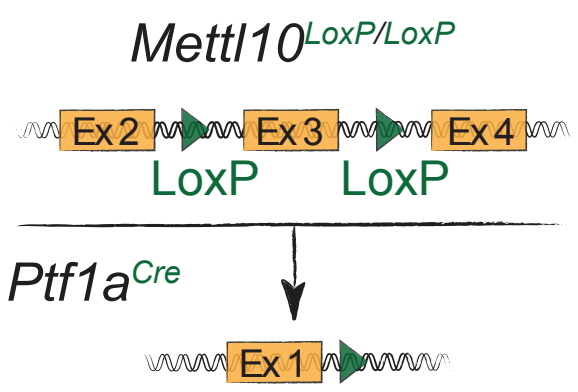

C

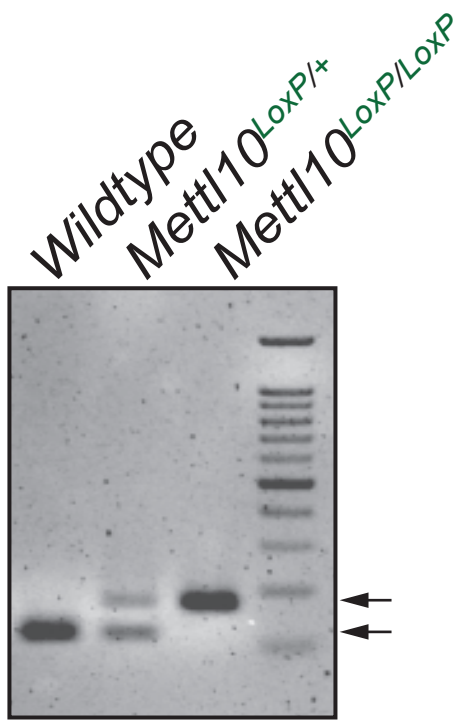

Supplement: Supplementary file 1 [file mmc1.pdf]
